# Dermatologic Toxicities from Monoclonal Antibodies and Tyrosine Kinase Inhibitors against EGFR: Pathophysiology and Management

**DOI:** 10.1155/2012/351210

**Published:** 2012-09-11

**Authors:** Shaad E. Abdullah, Missak Haigentz, Bilal Piperdi

**Affiliations:** Division of Oncology, Department of Medicine, Montefiore Medical Center, Albert Einstein College of Medicine, Bronx, New york, NY 10461, USA

## Abstract

Epidermal growth factor receptor (EGFR) inhibition has now been well established as an effective treatment for various cancers. The EGFR belongs to the ErbB family of tyrosine kinase receptors which regulate tumor cell differentiation, survival and proliferation. Activation of EGFR drives tumorigenesis in lung, head and neck, colorectal and pancreatic cancers. Irrespective of the type of cancer being treated and the mechanism by which tumor EGFR drives tumorigenesis, the major side effect of EGFR inhibition is a papulopustular (also described as maculopapular or acneiform) rash which occurs in about two thirds of treated patients. Interestingly, this rash has been commonly correlated with better clinical outcomes (objective tumor response and patient survival). The pathophysiology of dermatological toxicity from EGFR inhibitors is an important area of clinical research, and the proper management of the rash is essential to increase the therapeutic index from this class of drugs. In this paper, we review the dermatologic toxicities associated with EGFR inhibitors with an emphasis on its pathophysiology and clinical management.

## 1. Introduction

Epidermal growth factor receptor (EGFR) inhibition has now been well established as an effective treatment for various cancers. EGFR belongs to a family (ErbB) of tyrosine kinase receptors which regulate tumor cell differentiation, survival, and proliferation. EGFR drives tumorigenesis as a result of activating mutations in adenocarcinoma of the lung and by less defined mechanisms of pathway activation (increased expression of receptors or ligands) in other malignancies such as head and neck cancer, colorectal cancer, squamous cell carcinoma of the lung, and pancreatic cancer [1].Best responses and clinical benefit have been seen in malignancies with EGFR activating mutations but clinical benefit has also been observed in conditions where the pathway is not activated as a result of EGFR mutations.

Irrespective of the type of cancer being treated and the mechanism by which tumor EGFR drives tumorigenesis, the major side effect of EGFR inhibition is a papulopustular (also described as maculopapular or acneiform) rash which occurs [1] in about two thirds of the patients. When severe (grade 3, in about 10% of the patients), it often leads to treatment discontinuation. In a larger number of patients, it affects quality of life affecting compliance and often results in treatment dose adjustments or temporary interruptions [2–4]. Different reports suggest that dose modifications or interruptions as a result of skin toxicity occur as often as about 30% of patients [5, 6]. Understanding the pathophysiology and management of dermatological toxicity from EGFR inhibitors is an important area of clinical research, and the proper management of the rash is essential to increase the therapeutic index from this class of drugs. There is no general consensus regarding the treatment of the rash. Several recent trials have evaluated empiric interventions and attempts have been made to establish guidelines [7–10]. Interestingly, when the relationship has been studied, the rash has been uniformly correlated with better clinical outcomes (objective tumor response and patient survival) both when the anti-EGFR agents are used as single agents or in combination with chemotherapy [11–16]. In this paper, we will review the dermatologic toxicities associated with EGFR inhibitors with emphasis on pathophysiology of the rash and its management.

## 2. Epidermal Growth Factor Receptor and Pathway

The erbB oncogenes encode the HER family of tyrosine kinase receptors, which namely consists of EGFR or HER1, HER2, HER3, and HER4. All members of the HER family consist of a receptor which comprises of an extracellular site concerned with ligand binding, a hydrophobic transmembrane domain, and an intracellular tyrosine kinase domain. Ligands binding to the EGFR are namely the epidermal growth factor (EGF), amphiregulin, *β*-cellulin, epiregulin, Heparin-binding EGF-like ligand (HB-EGF), transforming growth factor alpha (TGF*α*), and others. Binding of the ligand to the extracellular domain of the receptor results in conformational change and subsequent dimerization of the receptor with other HER family receptors. After dimerization, the internal tyrosine kinase is activated resulting in a downstream signaling cascade involving various pathways such as the PI3K/AKT, Ras/Raf/MAPK, and so forth. These activated signaling pathways result in interaction with essential cellular processes such as inhibition of apoptosis, gene transcription/translation, cell cycle progression, proliferation, and even processes involved with glucose metabolism [17, 18]. 

## 3. EGFR Inhibitors and Cancer

The EGFR is expressed in many different cell types in normal tissues, such as epithelial tissue, skin, hair follicles, and the gastrointestinal tract. In malignancy, however, dysregulation or overexpression of the receptor can occur leading to evasion from apoptosis, proliferation, invasion, metastases, and tumor induced angiogenesis [19–21]. Treatment options have become available over the years which inhibit the receptor or the downstream pathway, either by using monoclonal antibodies (MAbs) binding to the extracellular domain, small molecule tyrosine kinase inhibitors (TKIs) which bind to the intracellular domain, antisense oligonucleotides which decrease the expression of EGFR, immunotoxins which deliver toxins by targeting the receptor, and direct inhibitors of downstream signaling. The first EGFR inhibitor (EGFRI) to become widely available for clinical use was cetuximab (Erbitux), a chimeric human/murine immunoglobulin (IgG1) monoclonal antibody which binds to EGFR which higher affinity than EGF or TGF*α* [22]. Cetuximab was first approved by the US FDA in 2004 in combination with irinotecan or as a single agent in patients unable to tolerate irinotecan for colorectal cancer. In 2006, cetuximab was approved for the treatment of squamous cell carcinoma of the head and neck in combination with radiation therapy or as a single agent in patients who had received cisplatin previously, while another monoclonal but fully humanized antibody panitumumab was approved for colorectal cancer in 2007 for metastatic disease. Available small molecule EGFR tyrosine kinase inhibitors are gefitinib (Iressa) and erlotinib (Tarceva) for patients with metastatic lung cancer.

## 4. EGFRI-Associated Rash and Pathophysiology

Dermatologic toxicities are the most common side effects associated with anti-EGFR therapy. The most common dermatologic toxicity resulting from EGFRI treatment is papulopustular eruption, also called acneiform rash. Additional toxicities include nail changes, hair changes, ocular changes, pruritis, xerosis, and photosensitivity or erythema. EGFR inhibitor-related rash occurs very frequently in the prescribed patients, usually starting within two to three days following initiation of EGFRI treatment, and worsen within one to three weeks. Although not life threatening, the rash can be significant in causing impairment in quality of life. Not only can the rash cause discomfort but also occur in areas such as the face were it can be cosmetically and emotionally detrimental for the patient. 

EGFR protein is normally expressed in a wide variety of tissues, including the normal epidermis and follicular keratinocytes in the basal layer, the outer root sheath of hair follicles, and various other cells of the dermal connective tissue system. Approximately 40,000 to 100,000 receptors are reported to be expressed on the cell surface of normal tissues [23], while the number can reach around 2 million receptors per cell in malignancy [24, 25]. EGFR takes part in the various essential stages of epithelial maintenance by causing epidermal growth, differentiation, taking part in wound healing and keratinocyte migration. Hence, under EGFR inhibition, there is abnormal upkeep of the epithelium resulting in altered maturation and neutrophilic suppurative infiltrate in the dermis, particularly involving the follicular infundibula. The follicles are frequently enlarged in size and sometimes obstructed by excess keratinocyte plugs, while the sebaceous glands are usually not affected. No consistent changes in the cutaneous microflora have been found [26]. 

In animal models, inhibition of EGFR blocks downstreams signaling pathways and prevents keratinocytes from maturing properly as they migrate to the outer stratum corneum [27]. This results in the thinning of the outermost layers of the epidermis and corneal layers, and the subsequent loss of the skin's protective barrier function results in increased sensitivity to UV radiation damage. 

The EGFRI-associated rash appears to be clinically more severe with the use of monoclonal antibodies compared to the EGFR tyrosine kinase inhibitors (Table 3) [28]. Of note, the severity of rash has not always been directly correlated with the degree of EGFR inhibition. In a set of studies in patients treated with erlotinib, the skin inflammatory reaction was found to be independent of the degree of EGFR inhibition, and the cellular response was found to be mediated by TRAIL-positive mononuclear cells initially, and later by polymorphonuclear cells when superinfection from the skin occurs [29]. There is very little information available regarding the clinical or molecular predictors of rash severity from EGFRI. Although EGFR activating mutations in tumors have been predictive for clinical benefit to EGFR targeting agents (specifically TKIs) [30], these somatic mutations are present in only the tumor and therefore cannot account for the positive association of skin rash and clinical benefit with these agents. Research attention has recently focused on EGFR gene polymorphisms, present in both skin and tumor tissues. Various pharmacodynamic and pharmacogenomics models have been studied where EGFR polymorphisms have positively correlated with appearance of skin rash in patients, including downstream polymorphisms such as of the PI3 kinase pathway [31–34]. It has been postulated that these polymorphisms play a role in the positive overall survival correlation of the skin rash. The host susceptibility to respond in the form of an inflammatory reaction to EGFR inhibition may play an important role in the pathogenesis of EGFRI-associated rash. The role of host immune system in the pathogenesis of EGFRI-related dermatologic toxicities is poorly understood.

The combination of these inflammatory events leads to the eruption of varying degrees from mild to severe with complicated superinfection since the innate barrier system of the skin is compromised. The skin can become sensitive to UV light, while pruritus, discomfort, irritation, skin flaking and, associated hail and nail changes severely hamper patient quality of life. Emotional distress and also body image issues can also severely impact the patients' day-to-day life and compliance to therapy [35]. 

Interestingly, recent results from clinical trials involving nimotuzumab, a new anti-EGFR monoclonal antibody, have reported to have lesser or no skin toxicity which has been postulated to be secondary to selective inhibition for tumor cell EGFR phosphorylation only while sparing the skin [36–38].

## 5. Grading of EGFRI-Associated Rash

It is important to follow the patient closely after initiation of EGFR inhibitor therapy for the development of rash which usually develops at the first 1-2 weeks, peaks at 3-4 weeks on therapy, and then most of the time diminishes in intensity over the next couple of weeks but often persists in mild form throughout the course of therapy. Several grading criteria have been developed to judge the severity of rash. The primary goals of these grading criteria have been to develop a uniform, common terminology for assessment of rash severity and to help clinicians tailor therapy depending on the severity of the rash. 

The National Cancer Institute Common Toxicity Criteria for Adverse Events (CTCAE), version 4.0, allows for a quick severity estimation of skin toxicity reactions. It provides a clinical score with five grades of severity but heavily relies on the body surface area distribution which is not specific to EGFRI-associated rash, which is often limited to the face, scalp, chest, and upper back. Similarly, the sensitive dermatological skin score (WoMoScore) focuses on the long-term assessment of acneiform skin rashes [39]. The WoMoScore is calculated from body involvement, facial involvement, and clinical grading of erythema, papulation, pustulation, scaling, and crusts, providing a clinical score ranging from 0 to 100 (Table 4). Mild skin changes score up to 20, moderate cases range between 20 and 40, whereas severe acneiform eruptions exceed a WoMoScore of 40. However, this scoring system also is not entirely specific to EGFRI-associated rash and is not always practical for routine use by clinicians and oncologists. 

Two other EGFRI-relevant grading systems have been proposed, the first by Pérez-Soler et al. in 2005 [40] (Table 1) which accurately allows physicians to tailor EGFRI therapy and to treat the rash proactively. Another class-specific grading system has been adapted recently by the Multinational Association of Supportive Care (MASCC skin toxicity group; Table 2) which also is helpful to the healthcare team in accurately grading the toxicity of EGFRI [41]. 

## 6. Other Dermatologic Toxicities Observed with EGFRI Therapy

 In addition to the common side effect of rash, therapy with EGFR inhibitors can also be associated with other dermatologic toxicities. These include nail, hair, and ocular changes, as well as xerosis, pruritis, and photosensitivity. Recognizing these side effects and educating, monitoring, and providing supportive care to patients will promote better compliance with the treatment and will help the patients with coping strategies for these toxicities.

### 6.1. Xerosis, Pruritis, and Photosensitivity

Patients on EGFRI therapy can have a scaly, dry, pruritic skin in varying degrees in any part of the body including ocular, perineal, and vaginal areas. Xerosis is defined as dry, flaking skin and is seen in about 35% of patients treated with EGFRIs. Pruritis is defined as an unpleasant sensation that leads to itching of the skin and occurs in response to the release of histamine. Although the xerosis and pruritis tend to occur together, some patients may experience dry skin only without pruritus. Itching as a result of dry skin and pruritis can often lead to superinfection, resulting in cellulitis and folliculitis. Xerosis can also lead to swelling and cracking of lips, mucosal irritation, erythema, and inflammation [42]. Patients treated with EGFRIs may also develop varying degrees of photosensitivity for which the mechanism is not currently understood. EGFRI-induced photosensitivity is usually characterized by erythema from UV-induced damage. Erythema may be painless or painful and associated with mild to severe desquamation [41]. 

### 6.2. Hair Changes

Alopecia and hirsutism have been reported with the use of EGFRI therapy. Usually, alopecia develops 2–4 months after initiation of EGFRI therapy in 5-6% of the patients, while hirsutism can occur after 1-2 months on EGFRI therapy. Hirsutism is usually characterized by causing hair curling and rigidity, facial hypertrichosis, and trichomegaly. The latter involving the eyelashes can lead to eye irritation and conjunctivitis [41, 43, 44]. 

### 6.3. Nail Changes

EGFRI therapy is also associated with paronychia which can occur in about 6–12% of patients and most commonly affects the nail bed of the great toe [45]. These nail changes lead to inflammation, tenderness, formation of pyogenic granuloma-type lesions, and fissuring of lateral nail folds or distal finger nail bed. When severe, nail extraction could be required. The nail changes usually develop after 2-3 months of therapy but can also be observed as late as 6 months on therapy and can persist a long time after the drug has been halted [42]. 

### 6.4. Ocular Changes

Corneal dryness and abrasions can occur on EGFRI therapy. Most ocular changes can occur early in the course of therapy, sometimes seen even in the first week or two of therapy and can significantly impair patient quality of life. The majority of ocular changes are secondary to involvement of the eyelids and include squamous blepharitis, trichomegaly, meibomitis, dysfunctional tear syndrome, and other miscellaneous changes such as iridocyclitis and corneal epithelial defects [46, 47].

## 7. Management of EGFRI-Associated Rash and Skin Changes

With increasing use of EGFR inhibitors in clinical practice, we will notice an increasing need to recognize dermatologic toxicities associated with these agents and understand their management. The proper evidence-based management of the dermatologic side effects will be of paramount importance to their optimal use across a variety of different cancers. The majority of the literature on managing EGFRI-related dermatologic toxicities includes descriptive and case studies describing skin treatment regimens for managing toxicities associated with EGFRI treatment. Several recent prospective studies have addressed and evaluated different interventions to mitigate or reduce the severity of EGFRI-associated skin rash. Though there are many avenues in terms of treating the side effects associated with EGFRI therapy they mainly are employed in two ways, either reactionary to treat the rash which has occurred or preemptive/prophylactically in advance to avoid progression to a stage which interferes with patients' quality of life and compliance with therapy. Attempts have also been made to develop a consensus on treating the rash associated with EGFRI [8, 9, 40, 48–51].

### 7.1. General Patient Recommendations

The proper patient education and understanding of the potential dermatological side effects from EGFRI is the essential cornerstone of its management. At the start of EGFRI treatment, the clinicians should inform their patients of potential EGFRI-related symptoms of dermatologic toxicities and possible lifestyle changes that may enhance their comfort. Information regarding a patient's occupation and previous skin conditions and sensitivities may help clinicians and providers design treatment strategies for individual patients that can help prevent or minimize EGFRI-related dermatologic toxicities. 

One such measure should be to advise avoidance of prolonged sun exposure. If sun exposure is expected while patient is on EGFRI therapy, then the use of sunscreen is recommended with an at least 15 sun protection factor (SPF) or higher cream which should be applied to exposed areas 1-2 hours prior to exposure and reapplied every 2 hours if out in the sun for an extended period [52]. Other general measures include use of moisturizing cream for dry skin, antihistamines for pruritis and proper nail care. Patients should be instructed to apply moisturizing cream at least twice daily using a thick alcohol-free emollient. Patients should avoid products that lead to drying of the skin such as alcohol-based products, soaps, and long hot showers which can also dry the skin [53]. Patients should be instructed to avoid the common topical antiacne agents such benzoyl peroxide, which has the potential to cause skin irritation and excessive drying.

## 8. Reactive Treatment Strategies for Skin Rash

The majority of studies and interventions have evaluated several treatment strategies for skin rash after it has developed from anti-EGFRI therapy. Guidelines established have investigated various modes of treatment tailored to severity of rash incorporating topical and systemic antibiotics, topical and systemic steroids, and immunosuppressives.

### 8.1. Topical Treatment

Topical steroids and antibiotics have shown benefit in treating rash and preventing superinfection. Medium to high potency topical steroid preparations (e.g., hydrocortisone 1% or 2.5%) have been employed to treat the rash after in vitro studies showed release of inflammatory cytokines following EGFRI therapy. The topical antibiotics commonly used are clindamycin, erythromycin, and metronidazole. European studies have suggested use of topical metronidazole and oral minocycline 100 mg one to two times daily as a treatment for acneiform rash [60, 61]. 

### 8.2. Oral Antibiotics

Several studies thus far have reported benefit from the reactive use of oral tetracycline-based antibiotics. Recent guidelines established by the MASCC skin toxicity study group [62] have graded preparations to the level of evidence available for their use in EGFRI-associated rash. In these recommendations, doxycycline 100 mg BID and minocycline 100 mg daily have been advised for systemic use. Doxycycline is a preferred agent in patients with renal insufficiency, while minocycline is less photosensitizing. 

In another study reported by Scope et al. from the Memorial Sloan Kettering Cancer Center, patients with metastatic colorectal cancer receiving cetuximab were randomly assigned to either oral minocycline with topical tazarotene (a retinoid that is part of the vitamin A family) or oral placebo with topical tazarotene to reduce or prevent cetuximab-related rash [7]. The primary endpoint was total facial lesion count. Forty-eight patients were randomly assigned to receive minocycline or oral placebo, along with daily tazarotene cream at the start of treatment. Results from the study indicated that during the first month of treatment, total facial lesion counts were less in patients who received minocycline, including less pruritus than the placebo arm (20% versus 50%, resp.; *P* = 0.05). Administration of tazarotene cream had no clinical benefit, and skin irritation was reported in one-third of patients. Results from this study have encouraged physicians and nurses to administer oral minocycline when treating patients with papulopustular eruptions associated with EGFRI treatment and to avoid use of retinoids. 

**Figure 1:**
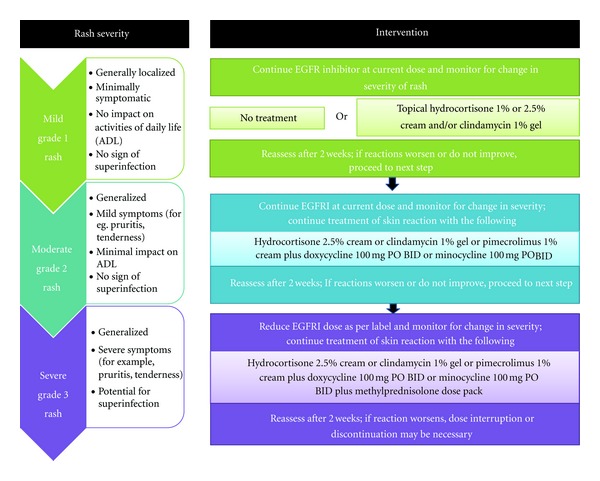
Treatment recommendations for EGFRI-associated rash. Adapted from Lynch et al. 2007 [45].

In another case study by Shu et al., treatment with oral minocycline was effective in the treatment of nail changes or paronychia elicited by EGFRI use. In summary, studies indicate a reactionary role for tetracycline-based oral antibiotics in the reactive management of skin rash associated with EGFRI. These agents have proven their efficacy in decreasing the number and extent of papulopustular eruptions in randomized trials with the use of oral minocycline [7], tetracycline [63], and doxycycline [8]. General treatment recommendations are summarized in Figure 1, which outlines the treatment protocol with increasing rash severity.

## 9. Preemptive or Prophylactic Rash Management

The skin toxicity evaluation protocol with panitumumab study (STEPP) compared in a randomized prospective manner the preemptive skin treatment protocol versus a reactionary skin treatment protocol in patients who were receiving panitumumab in combination with chemotherapy in patients with advanced colorectal cancers [8]. Patients on the reactive arm received treatment after the rash developed, while the pre-emptive group was assigned to receive daily skin therapy for a total of 6 weeks, starting 24 hours prior to the first dose of the EGFR inhibitor, panitumumab. This pre-emptive treatment protocol included sunscreen, moisturizer, hydrocortisone 1% cream, and oral doxycycline at 100 mg BID. When both arms were compared, the pre-emptive arm had a reduced incidence of grade 2 or greater rash by more than 50%, without any additional adverse effects of added therapy. There was also a significant delay in grade 2 or greater rash first occurrence in the pre-emptive arm. Also the Dermatology Life Quality Index (DLQI) showed that patient quality of life was less impaired in the pre-emptive arm. The median overall and progression-free survival was similar between the two groups, indicating that the preemptive treatment of skin rash had no adverse impact on the outcome from anti-EGFR therapy. This study makes a strong case of pre-emptive treatment of the rash but the use of multiple agents in the pre-emptive arm makes it difficult to assess which one of the pre-emptive treatments was most effective in creating this difference. 

Although a study reported by the researchers from the North Central Cancer Treatment Group, the role of oral tetracycline was examined in reducing the incidence of EGFRI-related rash [63, 64]. A total of 61 patients who received various anti-EGFR therapies were randomly assigned to receive either oral tetracycline (*n* = 31; 500 mg orally twice per day for 28 days) or placebo (*n* = 30). Tetracycline did not decrease the incidence of rash compared with placebo (70% and 76% of patients in the tetracycline and the placebo arm, respectively, developed papulo-pustular eruption). However, the severity of rash was significantly lower in patients receiving tetracycline. After four weeks of treatment, 17% of patients treated with tetracycline reported grade 2 papulopustular eruption compared to 55% in the placebo arm. Patients treated with tetracycline reported better quality of life scores (Skindex-16), including a decrease in skin burning and skin irritation.

## 10. Novel Agents in the Management of EGFRI-Associated Skin Rash

In addition to the preventive measures, topical and systemic treatments as described above, novel agents are being developed to reduce the severity and incidence of skin rash associated with EGFRI. One such agent is menadione, a synthetic prodrug of vitamin K [65]. When used topically/locally, menadione inhibits phosphatase and reduces the degree of EGFR inhibition induced by EGFRI on the skin, hence preventing the potential dermatological side effects from EGFRI's. It is currently undergoing phase I and II studies.

As a motivating factor, there is a clear positive correlation of efficacy and survival benefits with increasing severity of EGFRI-associated rash [1, 66]. This strong association of rash with survival has led to studies investigating a dose escalation protocol titrated to rash such as the EVEREST study involving cetuximab dose escalation in metastatic colon cancer [67]. The rash and survival correlation also makes one think of the possible immune mechanism behind the rash and the tumor response, where a possible increase in systemic cytokines/chemokines results in immunomodulation at the tumor level resulting in a better response. Various biochemical compounds are under development to actually augment this immune response to be used in conjunction with the EGFRI therapy [68], most prominently Imprime PGG (Biothera) has shown to improve survival even in the KRAS mutant colorectal cancer patients when used in combination with cetuximab (Erbitux) [69, 70].

## 11. Management of Radiation Dermatitis in Patients Receiving Anti-EGFR Monoclonal Antibodies

Monoclonal antibodies against EGFR are increasingly used with external beam radiotherapy in the treatment of head and neck cancer patients. By itself, radiation dermatitis is an expected side effect of radiation therapy secondary to direct injury to the skin tissue in the treatment field. Epidermal basal cells and connective tissue damage can occur in the first couple of weeks of radiotherapy which can be compounded by the use of EGFR inhibitor therapy. It is important that multidisciplinary approach is taken in treating this rash planning and delivery of radiotherapy are important and if rashes are severe then consideration should be made to adjust the radiotherapy dose. 

Cetuximab is the only targeted biological therapy approved in conjunction with radiotherapy for head and neck cancer. In the phase III trial that led to initial FDA approval for this indication, the frequency of observed radiation dermatitis (evaluated separately from acneiform dermatitis) was high (85–90%) in both cetuximab-treated and untreated patients [71]. However, high-grade radiation dermatitis has been reported from the addition of cetuximab to radiotherapy [72], and higher severe (grade 3-4) in-field dermatitis has been observed when cetuximab has been combined with cisplatin-based chemoradiotherapy [73]. Radiation dermatitis ranges from erythema and dry or wet desquamation to skin necrosis or ulceration of full thickness dermis with spontaneous bleeding from the involved site. Current recommendations as established by the MASCC skin toxicity study group [62] state that the irradiated area should be kept clean and dry, even when ulcerated. Gentle washing and drying of the skin within the radiation field have been shown to reduce the acute radiation-induced skin reaction [74, 75]. High-dose topical steroids have also been employed to treat radiation-induced dermatitis (mometasone, methylprednisolone, beclomethasone, and betamethasone topical preparations) [76, 77]. It is imperative that the healthcare team take a proactive approach in preventing this complication and prescribe appropriate therapy to treat the local area either by topical or systemic therapy described above in a timely manner. 

## 12. Conclusion

The EGFR inhibitors have become a valid antitumor therapy in many cancers and associated with their use is the commonly reported skin rash and other dermatologic toxicities. The skin toxicity can impair quality of life and interfere with patient's compliance with therapy. With the advent of such agents and future approvals of newer EGFRIs in development, it is becoming exceedingly important for the healthcare team to not only recognize and treat these adverse effects to improve individual patient's quality of life and compliance but employ appropriate adjustment of dose of medication when needed. With the exciting new developments in the field, we do hope to uncover the best mode of management of these adverse effects and at the same time prevent attenuation of the immune response which could benefit the patient in terms of treatment efficacy and survival outcomes associated with EGFRI therapy. 

## Figures and Tables

**Table 1:** Parmar et al. modified EGFRI rash grading system [31].

Grade 1	Macular or papular rash or erythema but no other associated symptoms	
Grade 2	Grade 2A	Grade 1 + pruritis or other symptoms which are tolerable
	Grade 2B	Grade + pruritis or other symptoms which interfere with daily life

Grade 3	Severe, generalised erythroderma, or macular, popular, or vesicular eruption	

Grade 4	Generalized exfoliative, ulcerative, or blistering skin toxicity	

**Table 2:** MASCC skin toxicity group proposed rash staging (papulopustular eruption, grading individually for face, scalp, chest, or back).

Grade 1	Grade 1A: papules or pustules <5; OR 1 area of erythema or edema <1 cm in size
	Grade 1B: papules or pustules <5; OR 1 area of erythema or edema <1 cm in size AND pain or pruritis

Grade 2	Grade 2A: papules or pustules 6–20; OR 2–5 areas of erythema or edema <1 cm in size
	Grade 2B: papules or pustules 6–20; OR 2–5 areas of erythema or edema <1 cm in size AND pain, pruritus, or effect on emotions or functioning

Grade 3	Grade 3A: papules or pustules >20; OR more than 5 areas of erythema or edema <1 cm in size
	Grade 3B: papules or pustules >20; OR more than 5 areas of erythema or edema <1 cm in size; AND pain, pruritus, or effect on emotions or functioning

**Table 3:** Dermatologic toxicity reported with single agent EGFR inhibitor therapy.

	Any grade (%)	Grade 3 and 4 (%)
Cetuximab [54, 55]	80–86	5–18
Panitumumab [56]	90	14
Erlotinib [11, 57]	75–79	5–10
Gefitinib [58]	62–75	Up to 4
Lapatinib [59]	27	1

**Table 4:** Definition of the score items of the WoMoScore.

*A* = body involvement	Extent of body lesions, 0–100%, according to the rule of nines.
*B* = facial involvement	Extent of lesions in the face, 0–100%
*C* = skin lesion score	Sum of:
	erythema intensity (0–3)
	erythema distribution (0–3)
	papulation (0–3)
	pustulation (0–3)
	scaling/crusts (0–3)

Calculation formula for final WoMoScore: WoMoScore = 1/4 *A* + 1/4 *B* + 10/3 *C*.
